# Bioactivation, Mutagenicity, DNA Damage, and Oxidative Stress Induced by 3,4-Dimethylaniline

**DOI:** 10.3390/biom14121562

**Published:** 2024-12-07

**Authors:** Mariam R. Habil, Raúl A. Salazar-González, Mark A. Doll, David W. Hein

**Affiliations:** Department of Pharmacology & Toxicology, University of Louisville School of Medicine, Louisville, KY 40202, USA; mariam.refaatzakyhabil@louisville.edu (M.R.H.); raul.salazar@dls.com (R.A.S.-G.); mark.doll@louisville.edu (M.A.D.)

**Keywords:** 3,4-dimethylaniline, NAT1, mutations, double strand breaks, genotoxicity

## Abstract

3,4-Dimethylaniline (3,4-DMA) is present in cigarette smoke and widely used as an intermediate in dyes, drugs, and pesticides. Nucleotide excision repair-deficient Chinese hamster ovary (CHO) cells stably transfected with human CYP1A2 and N-acetyltransferase 1 (NAT1) alleles: NAT1*4 (reference allele) or NAT1*14B (the most common variant allele) were utilized to assess 3,4-DMA *N*-acetylation and hypoxanthine phosphoribosyl transferase (HPRT) mutations, double-strand DNA breaks and reactive oxygen species (ROS). CHO cells expressing NAT1*4 exhibited significantly (*p* < 0.001) higher 3,4-DMA *N*-acetylation rates than CHO cells expressing NAT1*14B both in vitro and in situ. In CHO cells expressing CYP1A2 and NAT1, 3,4-DMA caused concentration-dependent increases in reactive oxygen species (ROS), double-stranded DNA damage, and HPRT mutations. CHO cells expressing NAT1*4 and NAT1*14B exhibited concentration-dependent increases in ROS following treatment with 3,4-DMA (linear trend *p* < 0.001 and *p* < 0.0001 for NAT1*4 and NAT1*14B, respectively) that were lower than in CHO cells expressing CYP1A2 alone. DNA damage and oxidative stress induced by 3,4-DMA did not differ significantly (*p* >0.05) between CHO cells expressing NAT1*4 and NAT1*14B. CHO cells expressing NAT1*14B showed higher HPRT mutants (*p* < 0.05) than CHO cells expressing NAT1*4. These findings confirm 3,4-DMA genotoxicity consistent with potential carcinogenicity.

## 1. Background

3,4-dimethylaniline (3,4-DMA; CAS No. 95-64-7; also identified as 3,4-xylidine) is an alkylaniline present in pharmaceuticals and personal care products, pesticides, tobacco smoke, and the dye industry [1]. Also, it is discharged in the wastewater of pharmaceutical and pesticide companies [2].

Bioactivation of alkylanilines may be mediated via *N*-hydroxylation by CYP1A2, followed by *O*-acetylation by *N*-acetyltransferase 1 (NAT1) [1,3,4,5,6]. Another bioactivation pathway occurs through oxidation to form quinone imines that can produce protein adducts by itself or oxidative DNA damage through reactive oxygens species (ROS) [1,4].

3,4-DMA is preferentially metabolized by *N*-acetyltransferase 1 (NAT1) [6]. The phenotype of one of the most common NAT1 variants (NAT1*14B) is substrate-dependent [7] and is associated with smoking-induced lung cancer [8] and urinary bladder cancer [9].

In previous studies, we used nucleotide excision repair (NER)-deficient Chinese hamster ovary cells (CHO) cells that express human CYP1A2 and NAT1 reference or variant alleles. A previous study reported that CHO cells transfected with NAT1*14B cells had higher covalent DNA adducts following treatment with 4-aminobiphenyl [7]. A more recent study showed that CHO cells transfected with CYP1A2 and NAT1 exhibited dose-dependent increases in reactive oxygen species, DNA damage, and mutations following treatment with benzidine [10]. In the current study, we used this model to investigate bioactivation, mutagenicity, DNA damage, and oxidative stress following exposure to 3,4-DMA.

## 2. Materials and Methods

### 2.1. Chinese Hamster Ovary (CHO) Cell Culture

The UV5-CHO cell line, a nucleotide excision repair-deficient derivative of the AA8 line [11], was obtained from the ATCC (Manassas, VA, USA, catalog number: CRL-1865). Since UV5-CHO lacks nucleotide excision repair due to a mutation in the *XPD (ERCC2)* gene, it is hypersensitive to bulky adduct mutagens and belongs to the excision repair cross-complementation group 2. Cells were grown in alpha-modified minimal essential medium (Cytiva, Logan, UT, USA, catalog number: SH30265.01) with L-glutamine, ribosides, and deoxyribosides supplemented with 10% fetal bovine serum (Hyclone, Logan, UT, USA, catalog number: SH30396.03), 100 units/mL penicillin, 100 micrograms/mL streptomycin (Hyclone, Logan, UT, USA, catalog number: SV30010), and 200 mM L-glutamine (Corning, VA, USA, catalog number: 25-005-CI) at 37 °C in 5% CO_2_. Media were supplemented with appropriate selective agents to maintain stable transfectants.

### 2.2. Construction and Characterization of UV5/CHO Cell Lines

The construction of UV5/1A2, UV5/1A2/NAT1*4, and UV5/1A2/NAT1*14B CHO cell lines were described and characterized previously [7]. Briefly, pFRT/lacZeo plasmid was transfected into nucleotide excision repair-deficient UV5 cell lines to generate a UV5 cell line containing a single integrated FRT site (UV5FRT). Purified human *NADPH cytochrome P450 reductase* (POR) and *CYP1A2* polymerase chain reaction (PCR) products were digested and ligated into similarly treated pIRES vector and transformed into DH5α competent cells. The pIRES plasmid containing cDNAs of human *CYP1A2* and POR was transfected into the newly established UV5FRT cell line. The open reading frames of NAT1*4 and NAT2*14B were amplified by PCR and inserted into the pcDNA5/FRT vector. The pcDNA5/FRT plasmid containing human NAT1*4 and NAT2*14B was co-transfected with pOG44, an Flp recombinase expression plasmid, into UV5/FRT/CYP1A2 cells. Integration of the pcDNA5/FRT construct into the FRT site was confirmed by PCR. The NAT1*4 and NAT2*14B-transfected cells were characterized for *N*-acetylation of PABA, a NAT1-selective substrate. NAT1 genotypes and deduced phenotypes were determined. Quantitative RT-PCR (RT-qPCR) assays were used to assess the relative amount of *CYP1A2* mRNA in cells in CHO cells. CYP1A2 protein expression was measured using in-cell Western protocol.

### 2.3. N-Acetyltransferase Assays In Vitro

CHO cell lysates containing 31–1000 µM 3,4-DMA (catalog: 126373, CAS number: 95-64-7 from Sigma Aldrich, St. Louis, MO, USA) and 300 µM acetyl coenzyme A (Sigma Aldrich, St. Louis, MO, USA) were incubated at 37 °C for 60 min. Reactions were terminated by the addition of 1/10 volume of 1 M acetic acid. Reaction tubes were centrifuged at 15,000× *g* for 10 min to precipitate protein. 3,4-DMA and acetyl 3,4-DMA (catalog: MS-20665, CAS number: 2198-54-1 from Cornwall, UK) were separated and measured by HPLC as described below. For all samples, protein concentrations of cell lysates were determined using the Bio-Rad protein assay kit (Bio-Rad, Hercules, CA, USA, catalog number: 5000006), and activity was calculated in nmoles of acetylated product/mL/min/mg protein.

### 2.4. N-Acetylation In Situ

CHO cells were thawed and transferred to 50 mL conical tubes containing 12 mL of alpha-modified minimal essential medium (Cytiva, Logan, UT, USA, catalog number: SH30265.01) with L-glutamine, ribosides, and deoxyribosides supplemented with 10% fetal bovine serum (Hyclone, Logan, UT, USA, catalog number: SH30396.03), 100 units/mL penicillin and 100 micrograms/mL streptomycin (Hyclone, Logan, UT, USA, catalog number: SV30010), and 200 mM L-glutamine (Corning, Manassas, VA, USA, catalog number: 25-005-CI). One mL of cell/media mixture was transferred to each well of 12 well plates to allow cells to attach for 24 h at 37 °C.

Following culture for 24 h, the cells were washed three times with 500 µL 1X PBS and replaced with media containing 3,4-DMA (31.3–1000 µM). Cells were incubated for 24 h, after which media was removed, and protein precipitated by addition of 1/10 volume of 1 M acetic acid. Media was centrifuged at 15,000× *g* for 10 min, and the supernatant was used to separate and quantitate all substrates and their acetylated products by high-performance liquid chromatography (HPLC), as described below. Cell number was determined after 24 h of incubation with each substrate, and activity was calculated in nmoles of acetylated product/24 h/million cells.

### 2.5. Identification and Separation of Substrates and Their N-Acetylated Products

The amount of acetyl 3,4-DMA produced was determined following separation and quantitation by HPLC using following separation and quantitation by HPLC subjected to a gradient of 100% 20 mM sodium perchlorate pH 2.5/0% acetonitrile for 5 min to 0% 20 mM sodium perchlorate pH 2.5/100% acetonitrile over 10 min, then to 100% 20 mM sodium perchlorate pH 2.5/0% acetonitrile over 5 min. Retention times for 3,4-DMA and acetyl-3,4-DMA were 8.08 and 10.1, respectively. The UV detector was set at 250 nm.

### 2.6. ROS

The intracellular ROS levels were evaluated using 2′,7′-dichlorofluorescein diacetate (DCF-DA) (Sigma-Aldrich, MO, USA, CAS number: 4091-99-0) with a slight modification of the method previously described [10]. In cells, this chemical is deacetylated to 2′,7′-dichlorodihydrofluorescein (H2DCF), which is further oxidized by ROS into a highly fluorescent 2′,7′-dichlorofluorescein (DCF). CHO cells were grown with selective agents in 10 cm plates, and 1 × 10^4^ cells were plated into black/clear bottom 96-well plates (Greiner Bio-One, Kremsmünster, AT, catalog number: 655986) and allowed to attach overnight. The next morning, media was removed, and attached cells were washed with PBS and replaced with fresh pre-warmed no phenol red α-Minimal Essential Medium (MEM) (catalog number: 41061029) FBS 5% containing 3,4-DMA (10–200 µM) for 24 h. Positive control wells were incubated with 1 mM H_2_O_2_ for 1 h.

### 2.7. γH2AX In-Cell Western Staining

DNA damage was assessed by a γH2AX in-cell Western staining protocol using slight modifications of a previously described method [10]. Cells were grown with selective agents in 10 cm plates, and 1 × 10^4^ cells were plated into black/clear bottom 96-well plates (Greiner Bio-One, Kremsmünster, Austria, catalog number: 655986) and allowed to attach overnight. The next morning, media was removed, and attached cells were washed with PBS and replaced with fresh pre-warmed no phenol red α-Minimal Essential Medium (MEM) (catalog number: 41061029), FBS 5% containing 3,4-DMA (50–500 µM) and incubated for 4 h. Media were removed, and γH2AX in-cell Western staining protocol was performed as previously described [10]. DNA and the γH2AX were simultaneously visualized using an Odyssey CLx imaging system (LI-COR, Lincoln, NE, USA) with the 680 nm fluorophore (red) and the 800 nm fluorophore (green). Relative fluorescent units for γH2AX per cell (as determined by γH2AX divided by DNA content) were divided by untreated cells.

### 2.8. Colony Formation and HPRT Mutations

Assays for cell survival and mutagenesis were slightly modified from methods previously described [10]. Briefly, cells were grown with selective agents in complete HAT-supplemented medium (Sigma-Aldrich, MO, USA, catalog number: H0262) for 12 doublings. Cells were plated at a density of 1 × 10^5^ cells/well in 6-well plates and incubated for 24 h, after which media was replaced, and the cells were treated for 48 h with 3,4-DMA (5–25 µM) dissolved in DMSO or vehicle control (0.5% DMSO). Survival was determined by a colony-forming assay and expressed as a percent of vehicle control. Colony efficiency dishes were seeded with 100 cells/well/6-well plate in triplicate and incubated for 6 days in complete media.

The remaining cells were replated and sub-cultured for 7 days. Then, dishes were seeded with 1 × 10^5^ cells/100 mm dish (5 replicates) and incubated for 7 days in complete medium containing 40 μM 6-thioguanine (6-TG) (Sigma-Aldrich, MO, USA, CAS number: 154-42-7). HPRT mutants were calculated per million cells and normalized to colony efficiency.

### 2.9. Statistical Analyses

Differences in *N*-acetylation rates and genotoxicity among CHO cells expressing different CYP1A2/NAT1 haplotypes were tested for significance using Student *t*-test, one-way ANOVA followed by a Tukey’s post hoc test, and two-way analysis of variance (ANOVA) followed by Bonferroni post hoc test. Michaelis–Menten kinetic constants were used to determine *K*_M_ and *V*_max_ for 3,4-DMA. All analyses were performed using GraphPad Prism 9, Inc., GraphPad, San Diego, CA, USA.

## 3. Results

### 3.1. In Vitro N-Acetylation

**Figure 1:**
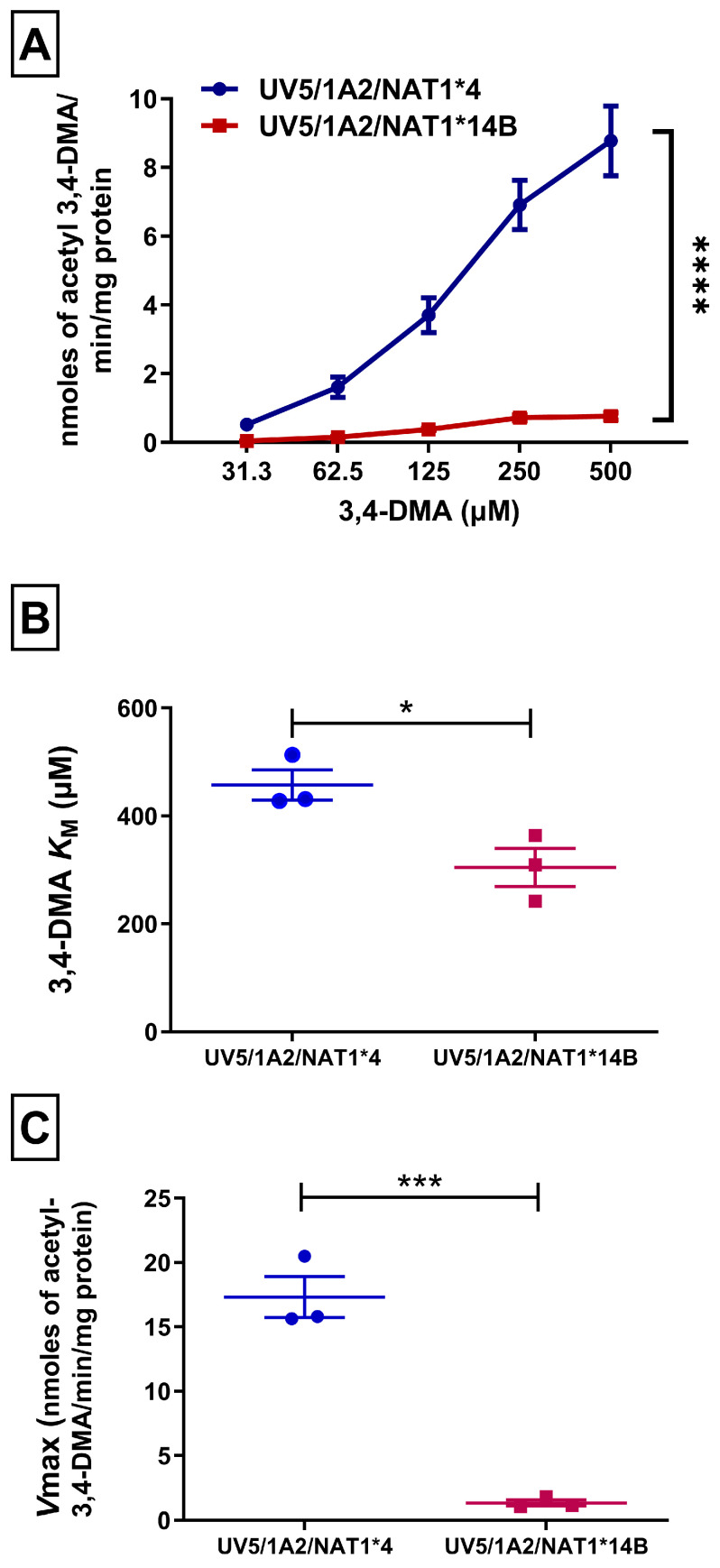
(**A**) *N*-acetylation rate of 3,4-DMA in vitro in CHO cell lysates using AcCoA 300 µM. Statistical significance was determined using two-way ANOVA followed by Bonferroni’s post hoc test. (**B**) NAT1*4 showed increased 3,4-DMA apparent *K*_M_ compared to NAT1*14B. (**C**) NAT1*4 showed higher *V*max compared to NAT1*14B. Michaelis–Menten equation was used to determine apparent *K*m and *V*max values. Unpaired T-test was used to test significance between the different alleles. Data illustrates mean ± SEM of three independent experiments. * *p* < 0.05, *** *p* < 0.001, **** *p* < 0.001.

NAT1 catalytic activity towards 3,4-DMA in the UV5/1A2/NAT1*4 cell line was significantly higher (*p* < 0.001) than in the UV5/1A2/NAT1*14B cell line (Figure 1A).

CHO cells were investigated for apparent *K*_M_ and *V*_max_ towards 3,4-DMA in the presence of 300 µM AcCoA. UV5/1A2/NAT1*14B cells exhibited higher affinity towards 3,4-DMA than UV5/1A2/NAT1*4 cells as indicated by lower apparent *K_M_* value (*p* < 0.05) (Figure 1B). The apparent *V*_max_ in UV5/1A2/NAT1*4 cells was about 13-fold greater (*p* < 0.001) than in UV5/1A2/NAT1*14B cells (Figure 1C).

### 3.2. In Situ N-Acetylation

**Figure 2:**
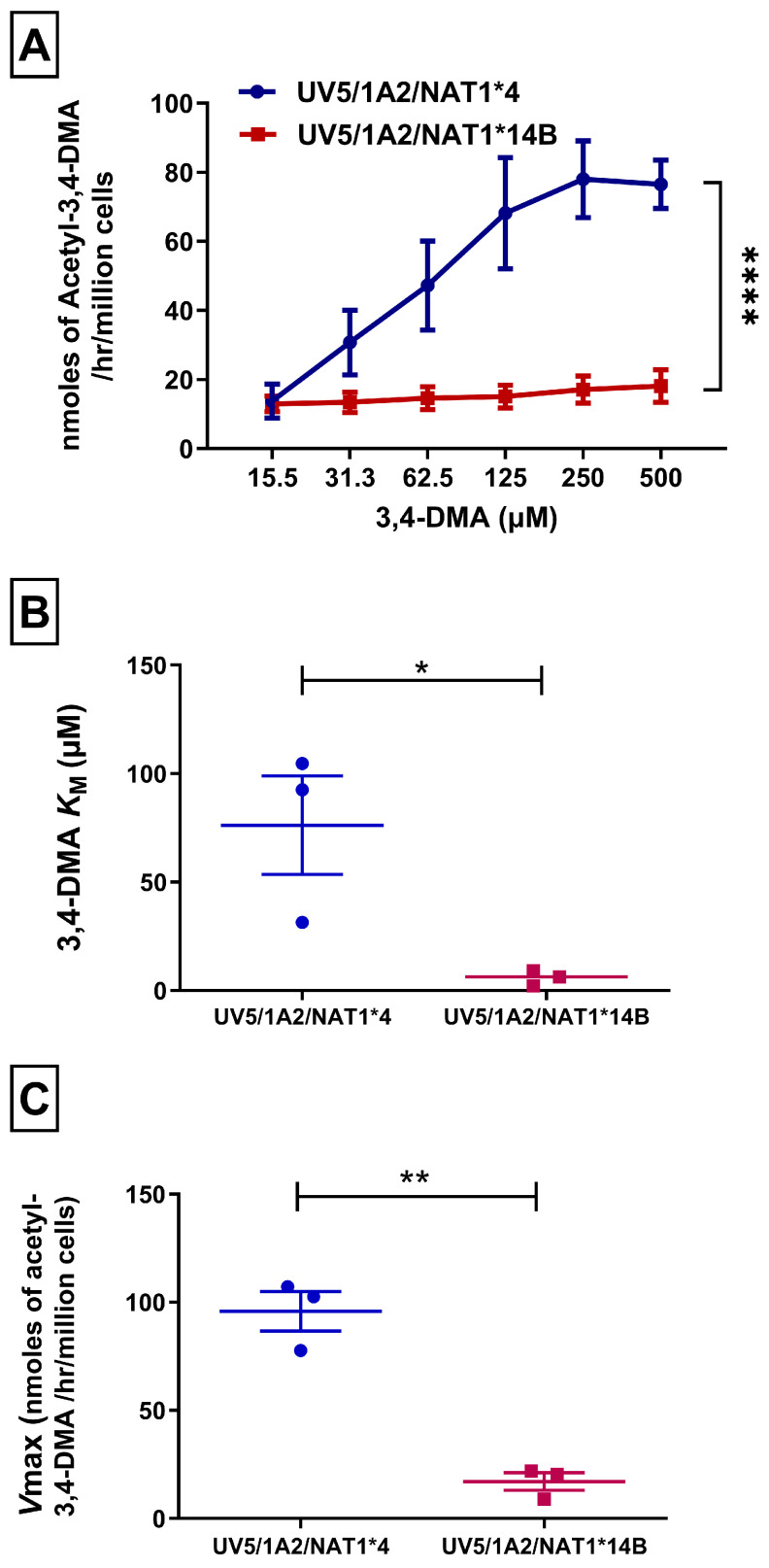
(**A**) *N*-acetylation rate of 3,4-DMA in situ in CHO cell lines. Statistical significance was determined using two-way ANOVA followed by Bonferroni’s post hoc test. (**B**) NAT1*4 showed increased 3,4-DMA apparent *K*_M_ compared to NAT1*14B. (**C**) NAT1*4 showed higher *V*max compared to NAT1*14B. Michaelis–Menten equation was used to determine apparent *K*_M_ and *V*max values. Unpaired T-test was used to test significance between the different alleles. Data illustrates mean ± SEM of three independent experiments * *p* < 0.05, ** *p* < 0.01, **** *p* < 0.0001.

In situ *N*-acetylation for 3,4-DMA in CHO cells was concentration-dependent (linear trend test showed *p* < 0.001). UV5/1A2/NAT1*4 cells exhibited significantly (*p* < 0.0001) higher *N*-acetylation of 3,4-DMA than UV5/1A2/NAT1*14B cells (Figure 2A).

Michaelis–Menten kinetics of UV5/1A2/NAT1*4 and UV5/1A2/NAT1*14B CHO cells to catalyze *N*-acetylation of 3,4-DMA was determined in situ. UV5/1A2/NAT1*14B cells had a higher affinity towards 3,4-DMA than UV5/1A2/NAT1*14B, as indicated by a significantly (*p* < 0.05) lower *K*_M_ value (about 15-fold) (Figure 2B). In addition, *V*_max_ in UV5/1A2/NAT1*4 cells was significantly (*p* < 0.01) 6-fold higher than in the UV5/1A2/NAT1*14B cells (Figure 2C).

### 3.3. ROS

**Figure 3:**
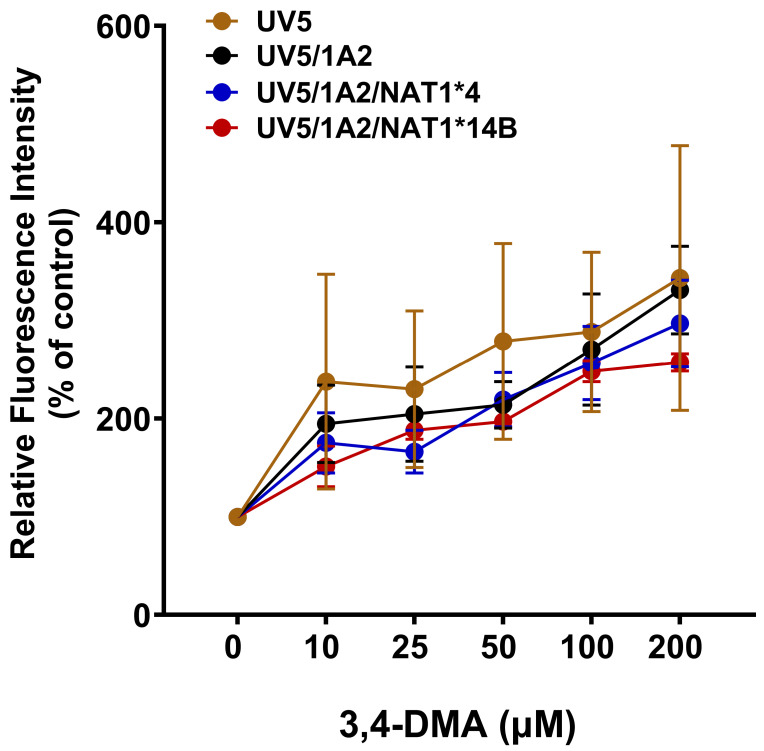
DCFDA assay in CHO cell lines showed concentration-dependent ROS generation in all CHO cell lines following treatment with 3,4-DMA (0–200 µM) for 24 h. Differences in ROS levels were slight and non-significant (*p* > 0.05) among the CHO cell lines but followed the pattern UV5 > UV5/1A2 > UV5/1A2/NAT1*4 > UV5/1A2/NAT1*14B. Data represents mean ± S.E.M. for three independent experiments.

Each of the CHO cell lines exhibited concentration-dependent increases in ROS following treatment with 3,4-DMA. Differences in ROS levels among the CHO cell lines were slight and non-significant (*p* > 0.05) but followed the pattern UV5 > UV5/1A2 > UV5/1A2/NAT1*4 > UV5/1A2/NAT1*14B (Figure 3).

### 3.4. γH2AX Signal

**Figure 4:**
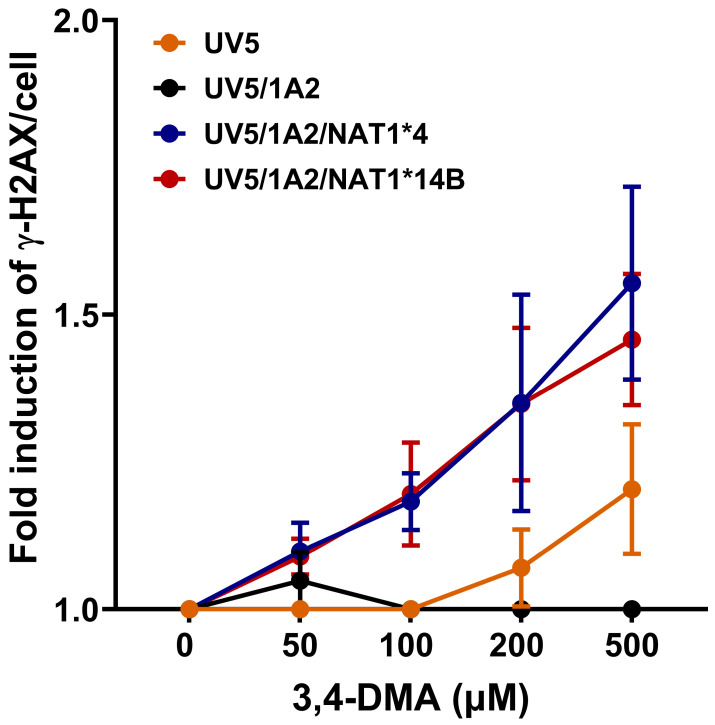
γ-H2AX in-cell Western assay in CHO cell lines. Data represents mean ± S.E.M. for three or four experiments. UV5/1A2/NAT1*4 and UV5/1A2/NAT1*14B CHO cells showed concentration-dependent increase in γH2AX signal (linear trend test *p* < 0.0001). There was no significant difference between UV5/1A2/NAT1*4 and UV5/1A2/NAT1*14B CHO cells (*p* > 0.05). Both UV5/1A2/NAT1*4 and UV5/1A2/NAT1*14B cells had significantly higher DNA damage compared to UV5 and UV5/1A2 cells (*p* < 0.05 for UV5, *p* < 0.001 and *p* < 0.01 for UV5/1A2).

Both UV5/1A2/NAT1*4 and UV5/1A2/NAT1*14B CHO cells showed a concentration-dependent increase in γH2AX signal (linear trend test *p* < 0.0001) following exposure to 3,4-DMA. The general pattern was UV5/1A2/NAT1*4 = UV5/1A2/NAT1*14B > UV5 > UV5/1A2. A significant difference between UV5/1A2/NAT1*4 and UV5/1A2/NAT1*14B CHO cells was not observed (*p* > 0.05) but both UV5/1A2/NAT1*4 and UV5/1A2/NAT1*14B cells had significantly higher DNA damage compared to UV5 and UV5/1A2 cells (*p* < 0.05 for UV5, *p* < 0.001 and *p* < 0.01 for UV5/1A2) (Figure 4).

### 3.5. HPRT Mutations

**Figure 5:**
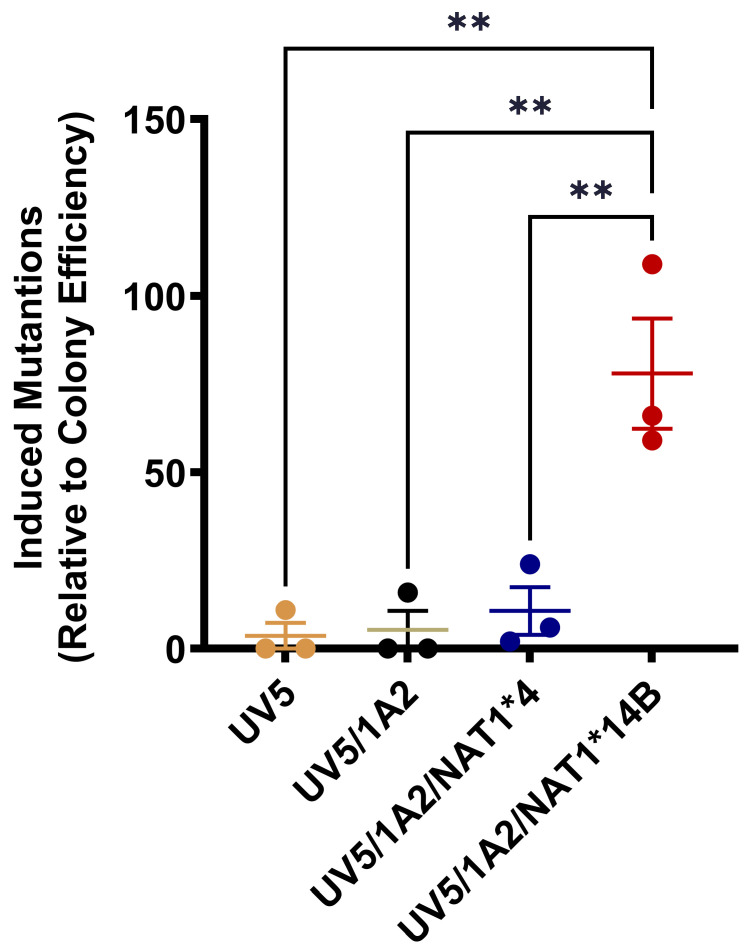
HPRT mutations following treatment with 3,4-DMA (5 µM) for 48 h. Data represents mean ± S.E.M. for three independent experiments. ** *p* < 0.01.

3,4-DMA (0–25 µM) treatment for 48 h did not significantly (*p* > 0.05) change the colony efficiency for any of the CHO cell lines. HPRT mutants were significantly higher (*p* < 0.001) in UV5/1A2/NAT1*14B cells than in any other CHO cell line (Figure 5).

## 4. Discussion

Although alkylanilines are highly prevalent in the environment, limited studies have explored alkylanilines as a class of human carcinogens. Exposures to aromatic amines [12], including alkylanilines such as 2,6-dimethylaniline (2,6-DMA), 3, 5-dimethylaniline (3,5-DMA), and 3-ethylaniline (3-EA) are associated with urinary bladder cancer [1,13]. High levels of 3,5-DMA- and 3-EA- hemoglobin adducts were detected in nonsmokers, suggesting the importance of other sources for alkylaniline exposure, such as dyes, motor fuels, pesticides, and drugs. Alkylanilines, including 3,4-DMA, are classified as category 3B carcinogens [14]. We used mammalian cells stably transfected with human CYP1A2 and NAT1 alleles to detect mutagenesis, double-strand DNA damage, and ROS generation following treatment with 3,4-DMA. CHO cells expressing human *CYP1A2* and NAT1*4 or NAT1*14B showed dose-dependent HPRT mutagenesis, γ-H2AX, and ROS consistent with 3,4-DMA genotoxicity.

NAT1*4 is the most common reference allele [15]. NAT1*14B is characterized by a single nucleotide polymorphism (SNP) G560A (rs4986782), resulting in an amino acid substitution R187Q. Computational homology modeling based on the NAT1 crystal structure indicates that the side chain of R187 is partially exposed to the domain II beta-barrel, the protein surface, and the active site pocket [16,17,18,19]. Homology modeling predicts that R187Q alters the binding of acetyl coenzyme A, active site acetylation, substrate specificity, and catalytic activity [15,16,17,18,19]. Previous studies have reported NAT1*14B to be associated with a reduced *N*-acetylation phenotype. Recombinant NAT1*14B expression in bacteria and yeast reduced *N*- and *O*-acetylation, NAT1 protein levels, and increased proteasomal degradation [20,21,22,23]. NAT1*14B is associated with reduced NAT1 catalytic activity and protein levels in peripheral blood mononuclear cells [24,25].

Previous studies have shown that the phenotype of NAT1*14B is substrate-dependent. NAT1*14 expressed in SV40-transformed African green monkey kidney (COS-1) cells resulted in decreased *V_max_* and decreased affinity towards p-aminobenzoic acid [26]. These findings were subsequently confirmed for NAT1*14 expressed in CHO cells [7,27]. In contrast, R187Q increased affinity towards 4-aminobiphenyl and N-hydroxy-4-aminobiphenyl [28].

In the current study, *N*-acetylation of 3,4-DMA in CHO cells transfected with NAT1*4 showed higher acetylation rates both in vitro and in situ than in CHO cells transfected with NAT1*14B. CHO cells transfected with NAT1*14B showed lower 3,4-DMA *K*_M_ values than NAT1*4, reflecting its higher affinity towards 3,4-DMA. This is consistent with previous findings that CHO cells expressing NAT1*14B showed higher affinity towards 4-aminobiphenyl and N-hydroxy-4-aminobiphenyl [7,28]. 3,4-DMA *K*_M_ comparisons between CHO cells expressing NAT1*4 and NAT1*14 were similar following measurements in vitro and in situ, but the lower 3,4-DMA *K*_M_ values determined in situ reflect a fixed AcCoA concentration of 300 µM for the in vitro assays, whereas CHO cells were not supplemented with AcCoA for the in situ assays.

The molecular mechanisms linking ROS to DNA damage and cancer were recently reviewed [29]. ROS generation is an important driving mechanism for the toxicity of many chemicals, including alkylanilines [30]. Investigations in cultured mammalian cells transfected with CYP1A2 and NAT2 concluded that the principal mechanism of 2,6-DMA genotoxicity is likely to be through redox cycling of intracellularly bound aminophenol/quinone imine structures to generate ROS rather than through formation of covalent DNA adducts [31]. Since 2,6-DMA is a very poor substrate for both NAT1 and NAT2 [6], the formation of covalent DNA adducts via *N*-hydroxylation followed by *O*-acetylation is highly unlikely. Previous studies showed that 2,4-DMA [32], 2,6-DMA [31,33], and 3,5-DMA [34] all induce ROS, which can then lead to oxidative DNA damage. 2,6-DMA is a very poor substrate for both NAT1 and NAT2, and 3,5-DMA is a preferential substrate for NAT2 versus NAT1 [6]. In our study, 3,4-DMA, which is a preferential substrate for NAT1 versus NAT2 [6], caused concentration-dependent increases in ROS in all the CHO cell lines. Differences in ROS levels among the CHO cell lines treated with 3,4-DMA were slight and non-significant (*p* > 0.05). Our findings showed 3,4-DMA induced lower ROS levels in CHO cells expressing NAT1*4 or NAT1*14B, suggesting that *N*-acetylation may serve as a protective mechanism against 3,4-DMA-induced oxidative stress. Our findings suggest that ROS formation is one of the factors underlying 3,4-DMA genotoxicity.

γ-H2AΧ has become a widely used biomarker for DNA double-strand breaks, which are associated with carcinogenesis [35,36,37]. In this study, we examined γ-H2AX generation induced by 3,4-DMA using an in-cell Western assay. 3,4-DMA generated γ-H2AΧ in a concentration-dependent manner in each of the CHO cell lines. This is consistent with a previous study reporting that 3,4-DMA induced DNA damage in chicken egg liver measured using comet assay [5]. CHO cells expressing NAT1 showed a higher γ-H2AΧ signal following treatment with 3,4-DMA compared to UV5 or UV5/1A2 consistent with DNA damage, including *N*-hydroxylation followed by *O*-acetylation catalyzed by NAT1. The higher γ-H2AΧ signal in CHO cells expressing NAT1 is consistent with this bioactivation pathway, although the results suggest DNA damage may also occur via alternative pathway(s).

Alkylanilines have been investigated for genotoxicity in Chinese hamster lung cells [38] and human urothelial cells [39]. Previous studies have shown inconsistent evidence of 3,4-DMA-induced genotoxicity. 3,4-DMA was a weak mutagen in reverse mutation test in bacteria with S9 activation [40]. A total of 98 chemicals were tested for the induction of chromosome aberration (CA), consisting of structural CA and polyploidy in Chinese hamster lung cells. Anilines tended to induce only structural CA. Among the anilines tested, 3,4-DMA did not induce structural chromosomal aberrations or polyploidy in Chinese hamster lung cells in the absence of the presence of exogenous rat liver S9 metabolic activation [38]. On the other hand, Kohara and workers reported positive comet results in the liver, lung, kidney, and bone marrow of mice treated with 3,4-DMA, while no micronuclei were observed in the bone marrow [41]. In the current study, HPRT mutants following treatment with 3,4-DMA were significantly higher in UV5/1A2/NAT1*14B cells than in any other CHO cell line. This suggests the importance of NAT1*14B in cancer risk following alkylaniline exposures, especially for those that are preferential substrates for NAT1, such as 3,4-DMA.

## 5. Conclusions

The main objective of the current study was to assess whether 3,4-DMA was genotoxic in CHO cells expressing human *CYP1A2* and *NAT1*. 3,4-DMA caused dose-dependent increases in ROS, γ-H2AΧ signal, and HPRT mutations consistent with genotoxicity via oxidative damage and/or covalent DNA adduct formation. Higher levels of γ-H2AΧ signal and HPRT mutations following treatment with 3,4-DMA were observed in CHO cells expressing CYP1A2 and NAT1*14B than in CHO cells expressing *CYP1A2* and NAT1*4. This is consistent with previous results following benzidine treatment [10]. Since multiple pathways and mechanisms for mutagenesis by dimethylanilines have been described [4], additional experiments are necessary to address the mechanism(s) for the results observed.

## Data Availability

The original contributions presented in this study are included in the article. Further inquiries can be directed to the corresponding author.
